# Simulating Urban Growth Scenarios Based on Ecological Security Pattern: A Case Study in Quanzhou, China

**DOI:** 10.3390/ijerph17197282

**Published:** 2020-10-05

**Authors:** Xiaoyang Liu, Ming Wei, Jian Zeng

**Affiliations:** 1 School of Architecture, Tianjin University, Tianjin 300072, China; liuxiaoyang@tju.edu.cn; 2 School of Transportation Science and Engineering, Harbin Institute of Technology, Harbin 150090, China; 3 School of Earth and Environmental Sciences, The University of Queensland, Brisbane 4072, QLD, Australia

**Keywords:** ecological security pattern, SLEUTH model, urban growth simulation

## Abstract

In recent decades, the ecological security pattern (ESP) has drawn increasing scientific attention against the backdrop of rapid urbanization and worsening ecological environment. Despite numerous achievements in identifying and constructing the ecological security pattern, limited attention has been paid on applying ESP to predict urban growth. To bridge the research gap, this paper took Quanzhou, China as a study case and incorporated the identified ESP into an urban growth simulation with three distinct scenarios. Following the “ecological source–ecological corridor–ecological security pattern” paradigm, the ESP identification was carried out from four single aspects (i.e., water, geology, biodiversity, and recreation) into three levels (i.e., basic ESP, intermediate ESP, and optimal ESP). Grounded in an equally weighted superposition algorithm, the four single ESPs were combined as an integrated ESP (IESP) with three levels. Taking IESP as an exclusion element, urban growth simulation in 2030 was completed with thee SLEUTH model. Drawing on the three levels of IESP, our urban growth simulation contained three scenarios. In terms of urban sprawl distribution coupled with urban growth rate, an optimal urban growth scenario is recommended in this paper to balance both urban development and eco-environment protection. We argue that our ESP-based urban growth simulation results shed new light on predicting urban sprawl and have the potential to inform planners and policymakers to contribute to more environmentally-friendly urban development.

## 1. Introduction

Human activity and ecological environment are intrinsically linked. In recent decades, the rapid development of urbanization has induced a series of environmental problems including the overconsumption of natural resources, environmental degradation, air pollution, and soil erosion [1,2,3,4]. These environmental problems, in return, intensify the risk of natural disasters and jeopardize human well-being in a wide variety of aspects from health and safety to living standards [5,6]. Sustainable development entails a stable eco-environment and vibrant ecosystem service [4,7]. As such, to achieve the trade-off between economic development and eco-environment protection, the concept of ecological security was proposed in the early 1980s [8,9]. Since then, from national governments to worldwide organizations, plenty of strategies to maintain ecological security have been enacted and implemented [10,11,12].

So far, in terms of different research perspectives, there are numerous definitions of ecological security. For example, Ezeonu and Ezeonu [13] deemed that ecological security refers to the states in which the health and integrity of the Earth’s ecosystem are well conserved, protected, and restored. Pirages [14] argued that ecological security is the balance between human need and environmental affordability for sustainable development. Synthesizing the definitions of ecological security in previous scholarship, the core element of this concept is in relation to coordinating the relationship between the natural ecosystem and economic society [8,13,14,15,16,17]. On account of the importance of ecological security, a growing body of literature on identifying ecological security patterns (ESPs) has emerged. To date, press-state-response (PSR) theory and circuit theory are the two conceptual frameworks most applied in extant literature [2,3,4,5,8,12,18,19]. Grounded in RSP theory, researchers first identify the pressure indicators allied with pressure-bearing indicators, then apply mathematical modeling and landscape ecology methods such as the artificial neural network model, entropy matter–element model, and grey model to calculate the weight of each indicator’s influence and integrate the weighted indicators to identify the ESP [8,18,20,21,22]. With respect to the studies based on circuit theory, researchers mainly follow the “ecological sources–ecological corridors–ecological nodes–ecological security pattern” paradigm, in which a series of ecological and spatial analytical approaches such as resistance surface construction, overlay analysis, and network analysis could be applied to achieve the construction of ESP [1,2,3,4,5,10,19].

Despite a fruitful line of research in analyzing ESP, there seems to be a lack of ESP application, particularly in incorporating ESP into urban growth simulation [1,2,3,4,5,6,7,8,9,15,16,17,18,19,23,24,25]. On one hand, most ESP-related studies have focused on ESP identification and construction. The effects of those identified ESPs in influencing or guiding urban growth have been neglected in the existing scholarship. Therefore, our knowledge of ESPs is limited in the scope of the current situation rather than future planning. On the other hand, a series of state-of-the-art urban growth simulation models has been dedicated to conducting future urban planning, however, these simulation models fail to take the potential effects of ESPs into account. Consequently, ESP, as an important factor in balancing urban development and eco-environment protection, has been arguably yet to be fully considered in urban simulation and its potential effects on urban planning remain unclear.

On the basis of the mechanism of cellular automata, a diverse range of models have been developed and improved to simulate and predict future urban growth over the past few decades [23]. Among these, the SLEUTH model has been widely applied due to its comprehensive evaluation and high accuracy [23,24,25]. SLEUTH is an acronym made up of its input datasets from six dimensions, namely, slope, land use/land cover (LULC), exclusion, urban extent, transportation, and hill-shade. Utilizing the input datasets, the SLEUTH model sets out five growth parameters (i.e., diffusion, breed, spread, slope resistance, and road gravity) and four growth types (i.e., spontaneous growth, new spreading center growth, edge growth, and road-influenced growth) to refine the calibration and finalize the urban growth prediction [26,27]. In building the exclusion layer, water bodies, wetlands, and reserved forest have mostly been used in previous studies, while, factors related to ecological security are scarcely mentioned [23,24,25,26,28,29]. As a matter of fact, it is evident that ESP covers a much larger extent than the conservation area [10,11]. In light of the importance of ESP in achieving sustainable development [1,2,3,4,5,6,7,8,9,10,11,12,15,16,17,18,19], there is a compelling need to incorporate ESP in urban growth simulation. It follows that if we, as a society, call for more eco-friendly and sustainable urban development, then ESP is required in simulating urban growth as a key facet in better informing planners and policymakers in landscape design and urban planning.

To bridge the aforementioned research gap, this paper took Quanzhou, a famous coastal city in Southeast China, as the study context and employed a suite of analytical approaches to fulfil the following objectives: (1) to identify the ESP from a single aspect to integrated aspect; (2) to classify the ESP into three levels, namely, basic ESP, intermediate ESP, and optimal ESP; and (3) to simulate urban growth with the incorporation of ESP to provide recommendations for future urban planning in terms of the simulation results.

The rest of this paper is organized as follows. Section 2 outlines the study context allied with data sources. Section 3 presents the analytical approaches plus the process of the SLEUTH model calibration. Section 4 provides the three levels of ESP from the single aspect to integrated aspect and the ESP-based urban growth simulation results. Section 5 discusses the implications of the modeling results and Section 6 presents avenues for future research before drawing some concluding remarks.

## 2. Study Context and Data Sources

### 2.1. Study Context

**Figure 1:**
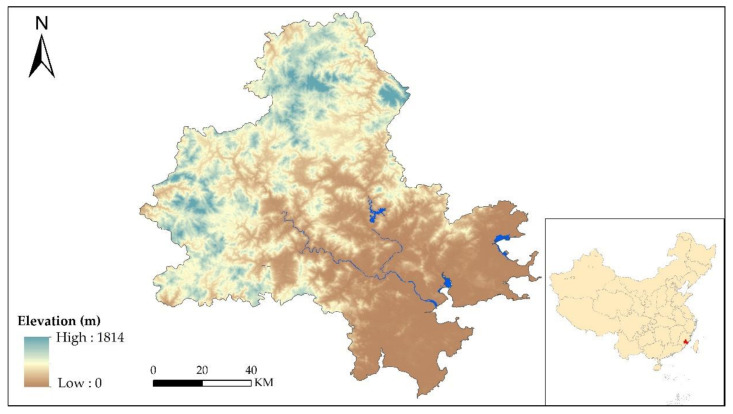
Location of Quanzhou.

Quanzhou (117°25′ E–119°05′ E, and 24°30′ N–25°56′ N) is situated in the eastern Fujian Province, China and is a prefecture-level port city known as the starting point of the Maritime Silk Road. In the humid subtropical climatic context, Quanzhou has rich water resources with an average annual rainfall up to 1400–2000 mm, and both the Jin and Luo Rivers flow into Quanzhou Bay in the Taiwan Strait (Figure 1). As one of the most developed cities in Fujian Province, Quanzhou has had the highest GDP (Gross Domestic Product) in the Fujian Province for 21 years [30]. Quanzhou is also the most populous city in Fujian Province, with over 8.5 million residents reported in the four districts, three county-level cities, four counties, and two special economic districts within Quanzhou [30].

### 2.2. Data

This study contained two major analytical components, namely, ESP identification and urban growth simulation. To accomplish these analyses, a wide range of datasets across transportation, land use, climatic context, and soil type were applied (Table 1). Our research data were mainly derived from official open sources and a series of satellite images. For example, land use data, the core dataset for both ESP identification and urban growth simulation, were obtained from the Resource and Environment Cloud Platform (http://www.resdc.cn/) across four periods (i.e., 2000, 2005, 2010, and 2015). Digital Elevation Model (DEM) data were achieved from the Geospatial Data Cloud (https://www.gscloud.cn/), and the corresponding information such as slope, curvature, and hill-shade was computed based on DEM data using an ArcGIS platform. Normalized difference vegetation index (NDVI), an indicator of vegetation greenness, was computed using Landsat 8 (https://earthexplorer.usgs.gov). It is noteworthy that in this study, nighttime light data were applied to revise the resistance surface in identifying the biodiversity ESP. More detailed data information is presented in the following table (Table 1).

## 3. Methodology

**Figure 2:**
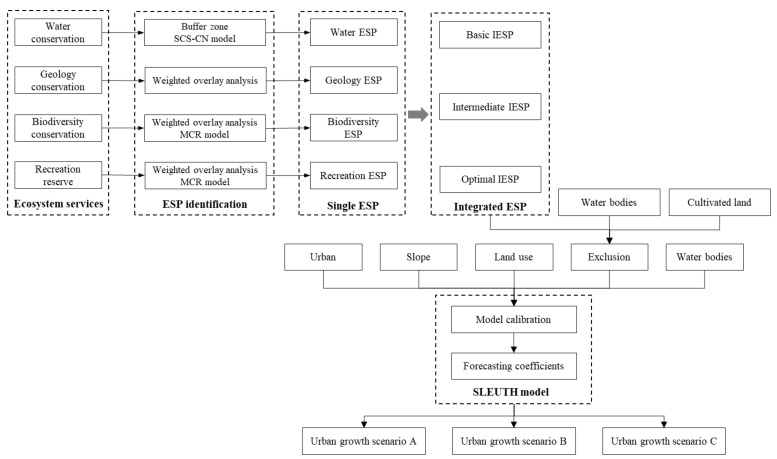
Methodological framework.

As discussed in Section 2.2, this study mainly comprises two analytical components, one identifies three levels of ESP in accordance with the “ecological sources–ecological corridor–ecological pattern” paradigm; the other uses the SLEUTH model to predict urban growth with the consideration of the identified ESPs. Figure 2 illustrates the completed methodological framework in this study and the following subsections outline the detailed analysis process to achieve the specific goals in ESP identification and SLEUTH model calibration.

### 3.1. ESP Identification and Construction

#### 3.1.1. Overlay Analysis

Overlay analysis is one of the most popular techniques in spatial analysis. Drawing on different spatial datasets equipped with diverse attribute information, overlay analysis creates an integrated layer by spatially combining information from different datasets. Spatial join is a core operation in overlay analysis, especially when layers are stored with different spatial resolutions or in different data types. By computing the information derived from different datasets, new attribute information can be added in the integrated layer. It is noteworthy that in adding new attribute information, certain selected factors can be weighted based on their importance to the overall goal.

In this study, overlay analysis was applied in identifying ESP for all four aspects and concerning identifying geology ESP, biodiversity ESP and recreation ESP, different weights were assigned in terms of the corresponding criteria (Figure 2).

#### 3.1.2. Soil Conservation Service Curve Number (SCS-CN) Model

Flood inundation area is a key aspect related to water ESP [19,31]. To incorporate this important information in water ESP identification and construction, the Soil Conservation Service Curve Number (SCS-CN) model was applied to estimate the surface runoff (Figure 2). Based on water balance and soil–cover complexes, the SCS-CN model assumes that the ratio of real soil retention after runoff to potential maximum retention is equal to the ratio of direct runoff to the maximum possible runoff. The detailed expression is as follows:

(1) $$\frac{Q}{P-I_{a}}=\frac{F}{S}$$

where *Q* is the gathered runoff (mm); *P* denotes rainfall (mm); *I_a_* is initial abstraction (mm); *F* is cumulative infiltration (mm); and *S* is the potential maximum retention (mm).

On account of the empirical findings, *I_a_* is equal to 0.2S. As such, the SCS-CN model can be rewritten as follows:

(2) $$Q=\left\{\begin{array}{c} \frac{{\left(P-0.2S\right)}^{2}}{P+0.8S},\;P\geq 0.2S\\ \;0,\;P<0.2S \end{array}\right.$$

In addition, according to the USDA (U.S. Department of Agriculture) handbook on hydrology section, the potential maximum retention can be calculated with the runoff curve number (CN), which varies by soil type [31]. The formula is expressed as follows:

(3) $$S=\frac{25400}{CN}-254$$

With the potential maximum retention (S) and gathered runoff (Q), the overall water volume (V) can be achieved as V = S × Q. Then, drawing on the possible rainfall occurrence, CN and DEM features, and the inundated area, a critical indicator of identifying waster ESP can thereby be computed.

#### 3.1.3. Minimum Cumulative Resistance (MCR) model

The minimum cumulative resistance (MCR) model was applied to extract the ecological corridor. The MCR model can build a resistance surface to estimate the difficulty of species diffusion from origin to destination. The mathematical expression is as follows:

(4) $$MCR=f_{min}\overset{i=m}{\underset{j=n}{\sum }}D_{ij}\times R_{ij}^{\prime }$$

where *MCR* represents the cumulative resistance value of a complete diffusion process; *D_ij_* denotes the distance from location *i* to location *j*; *R’_ij_* is the revised resistance value from location *i* to location *j*; and *f_min_* as a positive function examines the minimum value of all diffusion process. In this study, the MCR model is completed by the function of cost distance on the ArcGIS platform.

Drawing on the resistance surface, the ecological corridor can be extracted by the least cost path method on the ArcGIS platform. Based on the identified ecological sources, plus the extracted ecological corridors, the ecological security pattern can thereby be identified and established. In this study, the MCR model was applied for the identification of the biodiversity ESP and recreation ESP (Figure 2), and the original resistance surface was generated based on land use type.

#### 3.1.4. Resistance Surface Revision

Traditionally, the resistance surface construction only takes land use into account, without considering the potential influence of human activity on the process of species diffusion. To incorporate the effects of both human activity and natural environment, nighttime light data were employed in this study as a way to revise the resistance surface derived from land use. Given the different spatial resolution between land use (30 m) and nighttime data (100 m), the average nighttime light is resampled and recomputed for each land parcel of land use map. The detailed mathematical expression is as follows:

(5) $$NLI_{i}=\overset{n}{\underset{j=1}{\sum }}\left(\frac{Area_{i\left(j\right)}}{Area_{i}}\;\times \;NLI_{j}\right)$$

where *NLI_i_* is the average nighttime light for land parcel *i*, as every land parcel is divided into n pieces; *Area_i_* is the area of land parcel *i*; and Area_j_ is the area of piece *j* within land parcel *i*, and *NLI_j_* is the nighttime light for piece *j*. With the nighttime light for each land parcel computed, the resistance surface revision can be expressed as follows:

(6) $$R_{i}^{\prime }=\frac{NLI_{i}}{\left(\sum_{i=1}^{n}NLI_{a\left(i\right)}\right)/n}\;\times \;R_{i}$$

where *R_i_’* is the revised resistance value for land parcel *I*; *R_i_* is the original resistance value of land parcel *i*; *NLI_i_* is the average nighttime light for land parcel *i*; and (∑*n* *i* = 1 *NLI*a(*i*))/*n* represents the average nighttime light for all land parcels in land use type a. In this study, this revised resistance surface was applied in the MCR model to identify biodiversity ESP.

#### 3.1.5. Integrated ESP Construction

Following the “ecological sources–ecological corridor–ecological pattern” paradigm, the ESP was identified and constructed into three levels (i.e., basic ESP level, intermediate ESP level, and optimal ESP level) concerning four single aspects (i.e., water ESP, geology ESP, biodiversity ESP, and recreation ESP). To combine the four single ESPs into one integrated ESP (IESP), first, the three distinct ESP scenarios were assigned values of 1 for basic ESP level, 2 for the intermediate ESP level, and 3 for the optimal ESP level; second, drawing on the equally weighted superposition algorithm, the four identified ESPs were integrated with the maximum value among the four single ESPs. For example, a land parcel in basic water ESP (value 3), intermediate geology ESP (value 2), intermediate biodiversity ESP (value 2), and optimal recreation ESP (value 1) was identified as a basic IESP (value 3). The formula is expressed as follows:

(7) $$\mathrm{IESP}=\mathrm{Max}\left(ESP_{i}\right),\;i=3,2,1$$

where basic ESP level, intermediate ESP level and optimal ESP level were assigned values of 3, 2, 1, respectively. In this study, the integration model was completed using the mosaic function in ArcGIS raster analysis.

### 3.2. SLEUTH Model Calibration

The SLEUTH model requires input data from five aspects, namely, slope, land use, exclusion, urban extent, transportation, and hill-shade [32]. In this study, four land-use layers (i.e., land use map in 2000, 2005, 2010, and 2015), two road layers (i.e., road map in 2010 and 2015), one slope layer and one exclusion layer with three elements (i.e., IESP, water bodies, and cultivated land) were equipped to run the SLEUTH model. The SLEUTH model mainly contains two parts: calibration and simulation. Based on the historical data coupled with customized settings, the model was calibrated from coarse level, fine level to final level. In the process of the calibration, five controlling parameters were generated to determine the mechanism of urban growth (i.e., dispersion coefficient, breed coefficient, road-gravity coefficient, slope resistance coefficient, and spread coefficient). In terms of the five controlling parameters, four types of urban growth are predicted, namely, spontaneous growth, new spreading urban centers, edge growth around existing urban areas, and road-influenced growth [33]. To examine the accuracy of model calibration and attain a more reliable prediction result, the optimal SLEUTH metric (OSM) was employed to present the best goodness-of-fit:

(8) OSM=compare×pop×edges×clusters×slope×xmean×ymean

where compare is the ratio of simulated urban population over actual urban population; pop refers to the least square regression score for modeled urbanization; edge refers to the least square regression score for modeled urban edges count; cluster refers to the regression score for modeled urban clusters; slope indicates the terrain surface; xmean is average x values for modeled urbanized cells; and ymean is average y values for modeled urbanized cells.

Drawing on the OSM expression, the accuracy of urban growth simulation contains four aspects: the size of urban growth (compare and pop), the shape of urban growth (edges and clusters), the position of urban growth (xmean and ymean), and the terrain of the study area (slope). With all these parameters from controlling coefficients, growth types, and growth accuracy equipped, the Monte Carlo iteration was conducted to gradually narrow the range of growth coefficients and gain better simulation goodness.

In this study, based on historical data, Table 2 shows the results of the SLEUTH model calibration. Subsequently, in line with the growth coefficients in the final calibration, we examined the simulation accuracy for 2005, 2010, and 2015, respectively (Table 3). The high simulation accuracy proved that our model calibration was qualified to run the simulation process. It is noteworthy that with the consideration of the three levels of IESPs, our urban growth model also incorporated three scenarios (Figure 2). Table 4 shows the exclusion scheme of the three IESPs in the urban growth simulation.

BFC: Best Fit Coefficient, MCI: Monte Carlo Iterations, NI: Number of Iterations.

## 4. Results

### 4.1. ESP Identification and Construction

#### 4.1.1. Water ESP Identification

**Figure 3:**
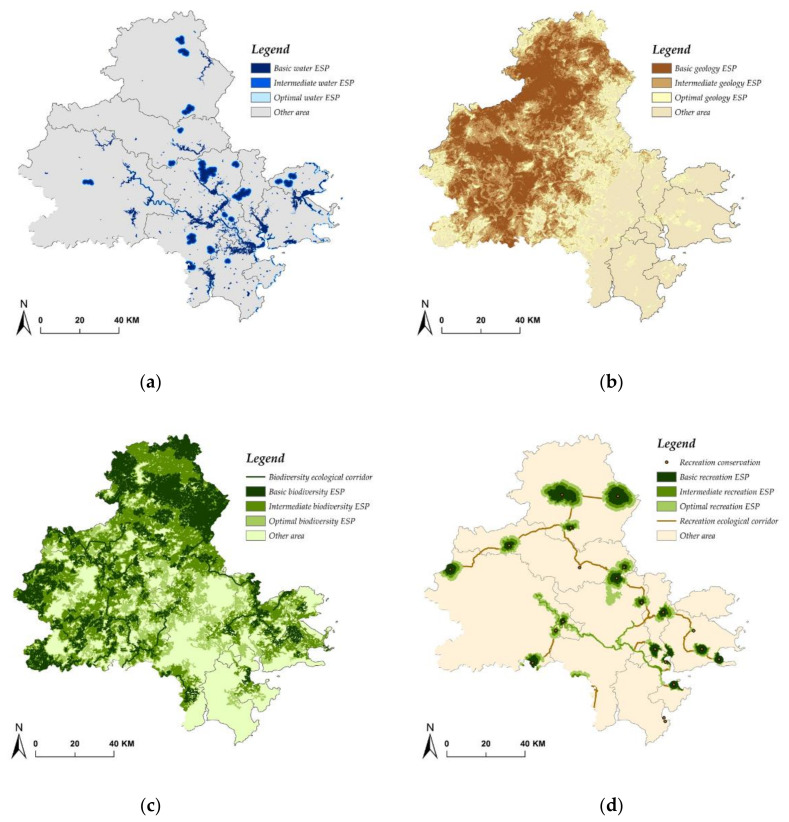
(**a**) Water ESP; (**b**) Geology ESP; (**c**) Biodiversity ESP; (**d**) Recreation ESP.

The ultimate purpose of water ESP identification and construction is to restore natural hydrological processes and avoid or mitigate flood risk. Thereby, drawing on previous scholarship [3,4,5], four evaluation factors were selected to comprehensively identify water ESP, namely, river and lake systems, surface water sources, flood storage area, and inundation area (Table 5). According to the criteria listed in Table 5, water ESP with three different levels was estimated by using the buffer zone and SCS–CN model (Figure 3a). As the water ESP in three different levels was spatially nested with similar shapes, the area became a major aspect of dissimilarity. Basic water ESP covers an area of 517 km^2^, intermediate water ESP covers 759 km^2^, and optimal water ESP covers 1115 km^2^.

#### 4.1.2. Geology ESP Identification

Considering the topographic characteristics and the influence of human activities, factors such as average annual rainfall, slope, elevation, surface curvature, soil type, NDVI, land use type, distance to major roads, and the number of geological hazards were selected to evaluate geology ESP in three levels, and their weights, sensitivity coupled with the corresponding criteria, were assigned in line with previous scholarship [1,4,5,18] and are presented in Table 6. The spatial distribution of geology ESP is illustrated in Figure 3b. It shows that the basic geology ESP is mainly located in the western part of the city, relatively distant from the built–up area, while optimal geology ESP covers a much larger area, except the western part, where some built–up adjacent areas are also highlighted.

#### 4.1.3. Biodiversity ESP Identification

**Figure 4:**
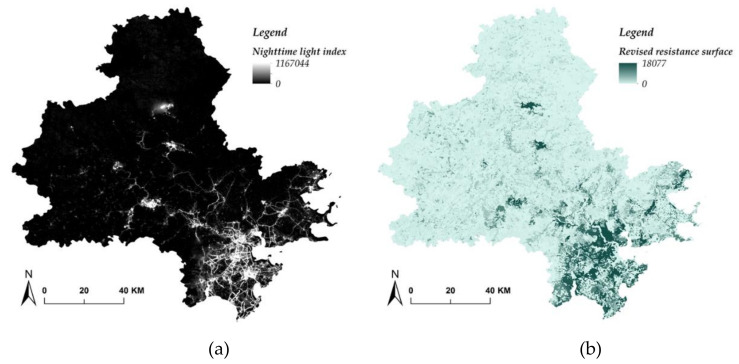
(**a**) Nighttime light in Quanzhou city; (**b**) Revised resistance surface.

According to the basics of habitat and the features of biological community, the ecological sources were evaluated by the criteria for biological habitat suitability (Table 7), in which both natural factors and human factors were incorporated and weighted. Drawing on land cover resistance (Table 8) and the MCR model, the resistance surface for biological migration was first built and subsequently revised by the nighttime light data (Figure 4). With the revised resistance surface (Figure 4b), the Jenks Natural Breaks Classification was applied to stratify the resistance value into four levels, namely, level 1 (0–271), level 2 (271–881), level 3 (881–1954), and level 4 (1954–18,077). In addition, in terms of the revised resistance surface and the identified ecological sources, the ecological corridors were extracted using the least–cost–path on the ArcGIS platform. As ecological sources, ecological corridors, and resistance surface were all estimated, the biodiversity ESP in three levels were identified in accordance with the criteria listed in Table 9. From Figure 3c, it is obvious that compared to the geology ESP, the spatial distribution of biodiversity ESP was less concentrated and much closer to the built–up area.

#### 4.1.4. Recreation ESP Identification

Similar to the process of biodiversity ESP identification, recreation ESP identification contains recreation sources identification, surface resistance establishment, and recreation corridor extraction. In the study area, recreation sources include two national parks, three provincial nature reserves, twelve provincial forest parks, one national scenic spot, and one provincial scenic spot. With respect to recreation corridors, 19 important corridors were extracted by applying the least–cost–path and gravity model [3,34]. Under the criteria in Table 10, the recreation ESP in three scenarios are identified in Figure 3d.

#### 4.1.5. Integrated ESP Identification

**Figure 5:**
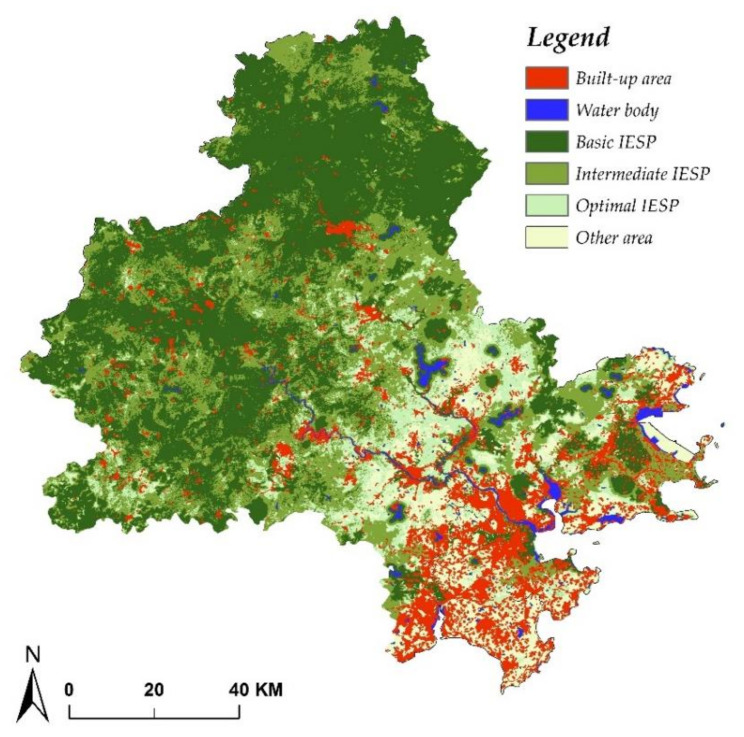
Integrated ESP with three different scenarios in Quanzhou city.

Combining the four single ESP with the principle of IESP (see Section 3.1.3), IESP in three basic, intermediate, and optimal scenarios were identified (Figure 5). Basic IESP represents the core areas of ecological restoration and ecological protection and plays an important role in maintaining eco–system service. The boundary of this area should form an ecological conservation redline and urban development should not invade into this area. Basic IESP covered an area of 4708 km^2^, accounting for 42.6% of the whole study area. The spatial distribution of basic IESP was mainly concentrated in the western mountainous area and water resource conservation, with the land cover of forest, wetlands, and grasslands. With regard to intermediate IESP, it is distributed as a surrounding belt of basic IESP with an area of 7817 km^2^ (70.8% of all study area). Given that the intermediate IESP encompasses peri–urban areas with high ecological significance, urban sprawl should be appropriately restricted in this intermediate IESP. Optimal IESP, with an area of 8964 km^2^ (81.2% of all study area), distributes the outer edge of built–up area. This area usually has less sensitivity to human activities and acts as a buffer zone between urban construction and ecological space. Under the primary goal of ecological protection, optimal IESP represents the ideal area for future development. In practical urban planning, optimal IESP can be moderately developed with conditional construction activities.

### 4.2. Urban Growth Modeling Results

**Figure 6:**
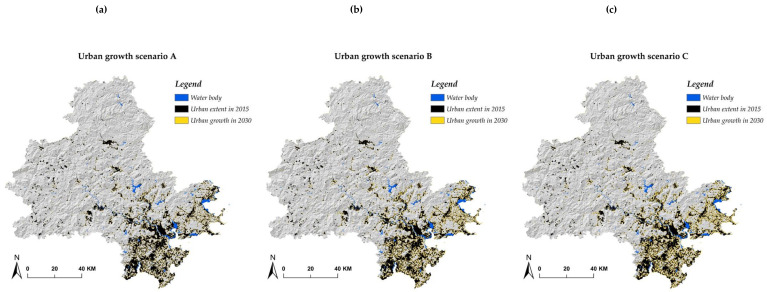
(**a**) Urban growth scenario A; (**b**) Urban growth scenario B; (**c**) Urban growth scenario C.

Incorporating IESP into exclusion layers of the SLEUTH model, the urban growth simulation from 2015–2030 also contained three distinct scenarios (Figure 6). Urban growth scenario A (Figure 6a) only takes basic IESP as the exclusion element, indicating apart from the basic IESP and the other two fixed exclusion layers (i.e., water body and cultivated land), all areas were available for urban sprawl. In comparison with urban growth scenario A, urban growth scenario B had relatively more restrictions, in addition to totally prohibited basic IESP, intermediate IESP could only be urbanized with a 30% area (Figure 6b). With respect to urban growth scenario C, it had the most restrictions on urbanization with the primary goal to restore the eco–environment (Figure 6c). Apart from 100% basic IESP and 70% intermediate IESP, 50% of optimal IESP was also excluded in urban development. From Table 11, it was observed that scenario A had the largest urban growth area and scenario C took the smallest urban sprawl. In terms of urban growth rate, it showed that all three urban growth scenarios had a lower rate than the historical record from 2000–2015. As for the spatial distribution, scenario C, in line with the principle of ecological priority, had the least invasion into the forest, grassland, and wetland. However, those urban growth areas were small and fragmented without spatial continuity, which might result in urban development being less efficient. Scenario A, following the principle of urban development, has shown a trend of integration of urban–rural development, while, at the price the large peri–urban eco–environment is occupied. In contrast with scenario A and scenario C, scenario B was shown to spatially satisfy the two–way demand of both eco–environment protection and urban development. In general, given the urban growth rate derived from the SLEUTH model simulation, taking ESP as a factor, it is shown to be an effective way to avoid disorderly urban sprawl and mitigate the ecological system pressure.

## 5. Discussion

The main contribution of this study was to incorporate multiple levels of ESPs to simulate urban growth into different scenarios. With this, one can quantitatively predict and evaluate the interaction between urban development and eco–environment protection, thereby shedding new light on a macroscopic planning scheme with the ultimate goal of safeguarding regional ecological security and satisfying human demand for ecosystem services. As we know, following the initiation of the reform and opening policy, China has experienced tremendous urbanization in recent decades, urban population percentage was up to over 60% in 2020 against 17.92% in 1978 [35]. On one hand, the booming population has brought dramatic ecological land transformed into construction land. On the other hand, along with the increasing human demand for ecological services, diverse pollution generated from urban population aggravates the pressure on the ecological system. Consequently, with declining ecological service, increasing urban environment issues such as urban heat islands have popped up and poses a danger to human health [36]. In this urgent situation, there has been growing scholarly attention to applying ESP in coordinating urban development and ecological protection [36]. On account of its strength in outlining spatial division, ESP is regarded as an effective tool to identify the important patches for ecological service where urban development should be restricted [1,2]. However, most of the ESP studies so far have remained at the stage of ‘how to identify the ESP and where the ESP is’; the application of ESP, particularly in simulating urban growth and guiding future urban planning, has rarely been investigated. Given the importance of ESP, this study proposes ESP–based urban growth simulation to address this research gap.

In an attempt to achieve sustainable urban development with ecological protection being the priority, in 2019, China established the national territory spatial planning system and stressed three red lines in urban construction including the red line of the permanent basic farmland, the red line of ecological protection, and the red line of urban growth boundary. In this ESP–based urban growth simulation, those red lines related patches and their potential evolution are shown in different scenarios. The red lines are rigid, which does not mean that the outer area is fixed and invariant. As a matter of fact, under the requirements of redlines, there are plenty of possibilities for the interaction between urban development and eco–environment protection. Drawing on the scenarios presented in this study, we can foresee the consequences in terms of different urban development strategies. These potential consequences would be valuable to suggest to urban planners ‘what can be achieved’ and ‘what should be avoided’. As such, the ESP-based urban growth simulation proposed in this study can provide a new understanding of guiding urban development in an orderly fashion and prevent uncontrolled urban sprawl. In addition, the paradigm proposed by this study for identifying ESP and simulating urban sprawl can easily be adapted to many other regions across the globe, especially for regions undergoing rapid urbanization in developing countries.

## 6. Conclusions

This paper applied the SLEUTH model to predict urban growth in three different scenarios with the consideration of ESP. Following the “ecological sources–ecological corridors–ecological security pattern” paradigm, ESP was respectively identified from four aspects, namely, water, biodiversity, geology, and recreation. Combining the four single ESPs, one integrated ESP was established and classified into basic IESP, intermediate IESP, and optimal IESP. Separately incorporating the three IESP levels as an exclusion element, urban growth simulation was carried out into three scenarios. The simulation results illustrate the possible urban growth in 2030 under China’s Ecological Redline Policy.

In terms of the different restrictions of basic IESP, intermediate IESP, and optimal IESP, three urban growth scenarios have different emphasis between eco-system protection and urban development. Urban growth scenario C was shown to cover large peri–urban eco–service areas with relatively intense urban sprawl. Urban growth scenario A was found to markedly maintain the ecological system, however, its urban sprawl was scattered and fragmented. Urban growth scenario B was shown to be an ideal scheme with reasonable urban development and ecological system safeguarded. Considering the significance of ESP and necessity of eco–environment protection, our results in the urban growth simulation were arguably important with the capacity of shedding new light on smart growth, urban growth boundary control, urban design, and master planning. This paper also presented a paradigm for future studies to simulate environmentally-friendly urban growth.

Despite the innovative design of this study, two aspects form the avenue for future studies. First, at the different stages of urban development, the influence of human activities on ecological services may not stay the same, and more sociodemographic factors such as income, GDP, and population can be considered in both ESP construction and urban growth. Second, in the context of rapid urbanization, urban growth has apparent features of complexity and uncertainty, so both natural factors and human factors should be spatiotemporally evaluated to capture the driving factors and achieve a better urban growth simulation. To conclude, from a particular view of eco–environment protection, we took Quanzhou, China, as the study case and simulated its urban growth in 2030. Our simulation results add to the growing body of both ESP construction and urban growth simulation, supplementing the evidence base for planners and policymakers to make better landscape designs and master planning.

## Figures and Tables

**Table 1:** Data information.

Data	Utility	Data Source
Land use	ESP identification; Urban growth simulation	Resource and Environment Cloud Platform http://www.resdc.cn/
Annual rainfall	Water & Geology ESP identification	National Meteorological Information Center http://data.cma.cn/; Local weather station records
NDVI	Biodiversity & Geology ESP identification	U.S. Geological Survey Landsat image
Slope	Water & Geology & Biodiversity ESP identification; Urban growth simulation	Calculated from DEM data; Geospatial Data Cloud https://www.gscloud.cn/
Elevation	Water & Geology & Biodiversity ESP identification	Derived from DEM data; Geospatial Data Cloud https://www.gscloud.cn/
Curvature	Geology ESP identification	Calculated from DEM data; Geospatial Data Cloud https://www.gscloud.cn/
Hill-shade	Urban growth simulation	Calculated from DEM data; Geospatial Data Cloud https://www.gscloud.cn/
Road	Geology & Biodiversity ESP identification; Urban growth simulation	National Geoinformation Service http://www.webmap.cn
Geological hazards	Geology ESP identification	Fujian Seismological Bureau; Fujian Water Conservancy Bureau
Soil type	Geology ESP identification	Fujian Agriculture Department
Nighttime light	Biodiversity ESP revision	Luojia-1 Satellite image http://www.hbeos.org.cn
Recreation resources	Recreation ESP identification	Ministry of Ecology and Environment of China; Fujian Forestry Bureau

**Table 2:** Calibration coefficients for the SLEUTH model.

Growth Coefficients	Coarse		Fine		Final		BFC
	MCI = 5		MCI = 7		MCI = 9		
	NI = 3163		NI = 7851		NI = 7796		
	OSM = 0.4679		OSM = 0.4723		OSM = 0.5034		
	Range	Step	Range	Step	Range	Step	
Dispersion	0–100	25	25–100	15	40–75	7	81
Breed	0–100	25	50–100	10	50–75	5	51
Road gravity	0–100	25	0–75	15	30–75	9	66
Slope	0–100	25	25–70	9	25–40	3	35
Spread	0–100	25	25–100	15	25–55	6	42

BFC: Best Fit Coefficient, MCI: Monte Carlo Iterations, NI: Number of Iterations.

**Table 3:** The SLEUTH model simulation accuracy.

Modelling Results	2005	2010	2015
Actual value (number of pixels)	64,455	106,264	127,652
Simulation value (number of pixels)	56,427	94,628	11,6457
Simulation accuracy (%)	87.54	89.05	91.23

**Table 4:** Exclusion probability of the selected layers in the three urban growth scenarios (%).

Exclusion Layers	Urban Growth Scenario A	Urban Growth Scenario B	Urban Growth Scenario C
Built–up area in 2015	0	0	0
Water body	100	100	100
Cultivated land	100	100	100
Basic IESP	100	100	100
Intermediate IESP	70	70	0
Optimal IESP	50	0	0

**Table 5:** Criteria for water ESP identification.

Evaluation Factor	Basic Water ESP	Intermediate Water ESP	Optimal Water ESP
Distance to river and lake (m)	≤50	50–150	150–500
Distance to surface water (m)	≤500	500–1000	1000–1500
Flood storage area (m^3^)	3rd level of water storage area	2nd level of water storage area	1st level of water storage area
Distance to inundation area (km^2^)	10–Year rain event	50–Year rain event	1000–Year rain event

**Table 6:** Criteria for geology ESP identification.

Evaluation Factor	Standardized Value					Weight
	No Impact Area	Optimal ESP	Intermediate ESP	Basic Security Pattern		
	Insensitive (1)	Mildly Sensitive (3)	Moderately Sensitive (5)	Sensitive (7)	Highly Sensitive (9)	
Average annual rainfall (mm)	<1300	1300–1400	1400–1500	1500–1600	>1600	0.15
Slope (°)	<5	5–15	15–25	25–35	>35	0.1
Elevation (m)	<200	200–500	500–800	800–1000	>1000	0.1
Curvature	−0.5–0.5	(−1.5, −0.5], [0.5–1.5)	(−2.5, −1.5], [1.5–2.5)	(−3.5, −2.5], [2.5–3.5)	(−∞,−3.5], [3.5, ∞)	0.1
Soil type	Paddy soil Calcareous soil	Saline soil Sandy soil Meadow soil Limestone soil	Lateritic soil	Yellow soil Yellow–red soil Rhogosol Lithosol	Purple soil	0.1
Normalized difference vegetation index (NDVI)	<0.55	0.4–0.55	0.25–0.4	0.1–0.25	<0.1	0.1
Land cover	Construction land Waterbody Wetland	Forest Natural grassland Improved grassland	Irrigable land Dryland Garden plot	Artificial grassland Wild grassland Saline–alkali land	Slash land Bare land Sandy land Gravel land	0.1
Distance to major road (m)	>5000	3000–5000	1500–3000	500–1500	<500	0.1
Geological hazards number (per 25 km^2^)	<2	2–4	4–6	6–8	>8	0.15

**Table 7:** Criteria for biological habitat suitability.

Evaluation Factor	Classification	Value	Weight
Land cover	Urban and other construction lands	0	0.35
	Rural residential land	1	
	Bare land	2	
	Lowly covered grassland	3	
	Dryland and medium covered grassland	5	
	Sparse forest and waterway	6	
	Shrub and highly covered grassland	7	
	Closed forest, lake, reservoir, wetland	8	
	Paddy filed and mudflats	10	
Elevation (m)	0–100	5	0.10
	100–800	10	
	800–1500	5	
	>1500	1	
Distance to water sources (m)	0–2000	6	0.25
	2000–7000	8	
	7000–15000	10	
	15000–30000	5	
	>30000	2	
Distance to Built–up area (m)	>6000	10	0.15
	4000–6000	5	
	2000–4000	3	
	0–2000	1	
	0	0	
Distance to road (m)	0–500	0	0.15
	500–1000	1	
	1000–2000	3	
	2000–4000	5	
	>4000	10	

**Table 8:** Land cover resistance for biodiversity and recreation ESP identification.

Land Cover	Resistance Coefficient	Land Cover	Resistance Coefficient
Closed forest	1	Mudflats	100
Shrub forest, highly covered grassland	10	Dryland	200
Medium covered grassland	20	Bare land and saline–alkali land	300
Sparse forest	30	Rural residential land	400
Paddy field	50	Urban land	500
Waterbody	50	Other construction lands	500

**Table 9:** Criteria for biodiversity ESP identification.

Evaluation Factor	Basic Biodiversity ESP	Intermediate Biodiversity ESP	Optimal Biodiversity ESP
Distance to biodiversity source (m)	0	0–200	200–300
MCR value	Level 1	Level 2	Level 3
Distance to biodiversity corridor (m)	<100	100–200	200–300

**Table 10:** Criteria for recreation ESP identification.

Evaluation Factor	Basic Recreation ESP	Intermediate Recreation ESP	Optimal Recreation ESP
Distance to recreation source (m)	0	0–200	200–300
MCR value	Level 1	Level 2	Level 3
Distance to recreation corridor (m)	<100	100–200	200–300

**Table 11:** Statistics of urban growth simulation.

Period	Urban Growth Scenarios	Urban Growth Area (km^2^)	Annual Urban Growth Rate (%)
2000–2015	Historical record	538.8	4.4%
2015–2030	Urban growth scenario A	750.5	3.6%
	Urban growth scenario B	677.7	3.3%
	Urban growth scenario C	498.4	2.5%
